# Transcriptional changes in the Japanese scallop (*Mizuhopecten yessoensis*) shellinfested by *Polydora* provide insights into the molecular mechanism of shell formation and immunomodulation

**DOI:** 10.1038/s41598-018-35749-x

**Published:** 2018-12-05

**Authors:** Junxia Mao, Wenjing Zhang, Xiaosen Zhang, Ying Tian, Xubo Wang, Zhenlin Hao, Yaqing Chang

**Affiliations:** Key Laboratory of Mariculture & Stock Enhancement in North China’s Sea, Ministry of Agriculture and Rural Affairs, Dalian Ocean University, Dalian, China

## Abstract

The Japanese scallop (*Mizuhopecten yessoensis*) is one of the most important aquaculture species in Asian countries; however, it has suffered severe infection by *Polydora* in northern China in recent years, causing great economic losses. The *Polydora* parasitizes the shell of scallops, badly destroying the shell’s structure. To investigate the molecular response mechanism of *M. yessoensis* to *Polydora* infestion, a comprehensive and niche-targeted cDNA sequence database for diseased scallops was constructed. Additionally, the transcriptional changes in the edge mantle, central mantle and hemocytes, tissues directly related to the disease, were first described in this study. The results showed that genes involved in shell formation and immunomodulation were significantly differentially expressed due to the infestation. Different transcriptional changes existed between the two mantle regions, indicating the different molecular functions likely responsible for the formation of different shell layers. The differential expression of genes for immune recognition, signal transduction and pathogen elimination presented an integrated immune response process in scallops. Moreover, neuromodulation and glycometabolism involved in the regulation process with relevant function significantly enriched. The study provides valuable information for mechanism study of shell formation and immunomodulation in scallops.

## Introduction

**Figure 1:**
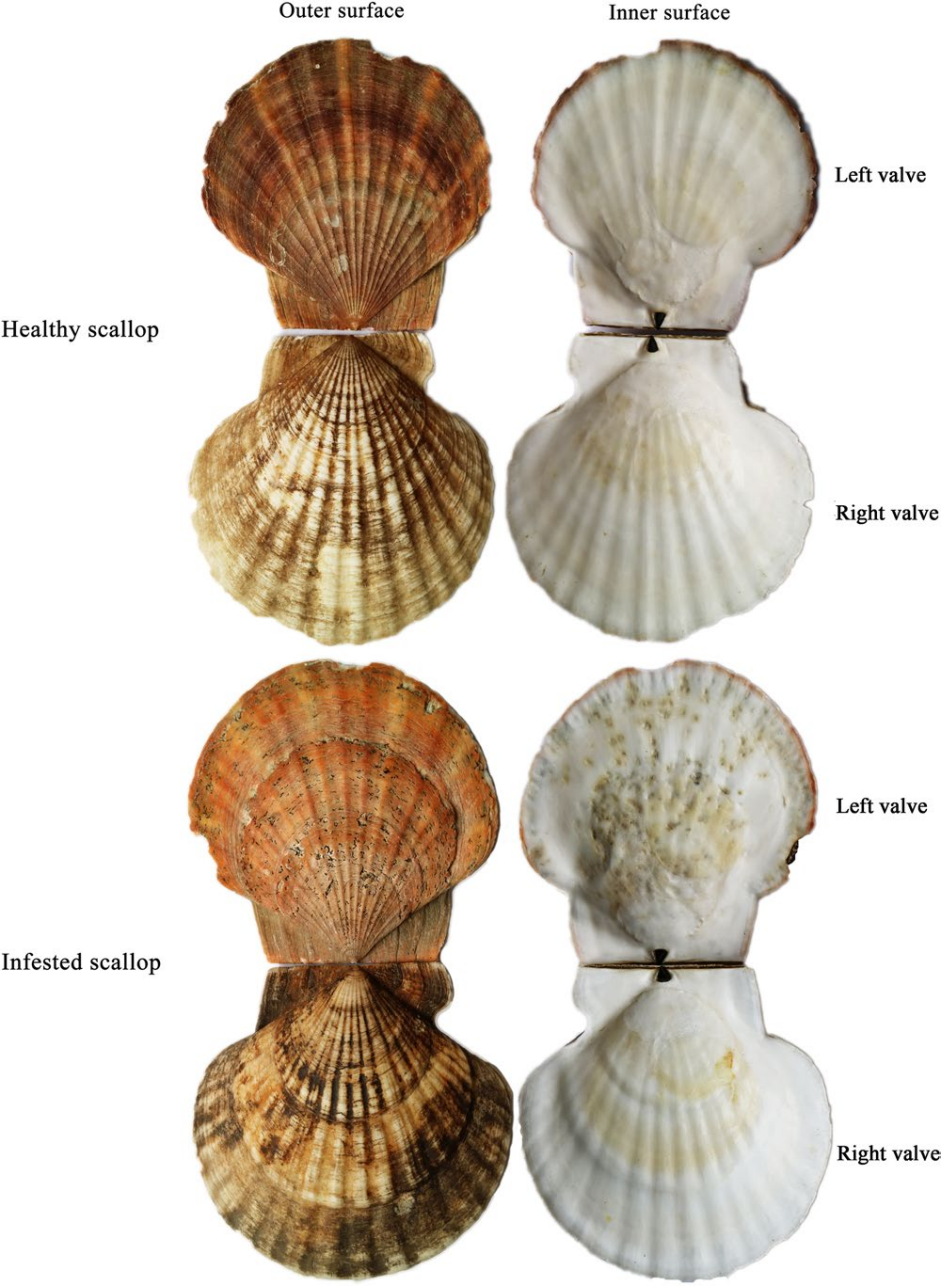
The outer and inner shell surfaces of the healthy (**A**) and infested by *Polydora* (**B**) *M. yessoensis*.

The Japanese scallop (*Mizuhopecten yessoensis*) is a large and old (dating back to ~350 Ma) group living on the cold and stable ocean bottoms of the northwestern Pacific Ocean. This species has been a major economic aquaculture species in Asian countries and is consumed worldwide^1^. However, the frequent outbreak of bacterial and parasitic diseases in *M. yessoensis* has caused severe economic losses. Spionid worms of the genus *Polydora*, which mainly inhabit the shells of many benthic bivalves^2–5^ including some commercially important species, have become a serious problem in recent scallop aquaculture. It was roughly estimated that over 90% of cultured *M. yessoensis* in the Dalian Zhangzidao Sea (Liaoning, China) had been infested by *Polydora* at various degrees, badly affecting the development of the scallop industry. *Polydora* mainly parasitize the left valve of the scallop by excavating U-type tubes, which badly damages the structure of the shells. To prevent further invasion, scallops accelerate the secretion of shell components to repair the damaged regions. As a result, many protuberances are formed on the inner surface of the shell (Fig. 1), which are signs of the shell repair process. When *Polydora* drill through the shell, they can directly infect the soft body (e.g. the mantle tissue) and the damaged shell exposes the scallop to various pathogens, activating the scallop’s immune modulation to combat the infection. At present, research about the *Polydora* disease mainly focuses on the biological characteristics and parasitic behaviour of *Polydora*^6–11^; however, the response of *M. yessoensis*, especially on the molecular level, has never been reported, limiting the understanding of disease resistance in scallops.

The mantle is the main tissue involved in the shell formation, located midway between the shell and visceral mass. Different mantle regions are responsible for the formation of different shell layers with the edge mantle being responsible for the periostracal and prismatic layerand the central mantle for the nacreous layer^12–14^. The resistance of *M. yessoensis* to the *Polydora* disease is characterized by the shell repair process with protuberance formation on the inner surface. Mantle tissue is therefore essential in the defense against *Polydora*. In recent years, the molecular function of mantle tissue has been extensively studied. With the prevalence of next-generation sequencing, transcriptomes of the mantle tissue have been sequenced in many mollusks, such as *Patella vulgate*^15^, *Mytilus edulis*^16^, *Hyriopsis cumingii*^17^, *Pinctada fucata*^18^, *P*. *margaritifera*^19,20^, *Chlamys farreri*^21^, and *M. yessoensis*^22,23^, to explore the mechanisms of shell formation. A wide variety of candidate genes involved in biomineralization have been identified, such as calcium-binding protein, carbohydrate-binding protein, glycoprotein, collagen, nacrein, perlucin and others, and their functions in shell formation have been partially characterized^15–23^. However, due to the extreme complexity of shell formation and the differences among species, our understanding is still fragmented and lacks consensus. In addition, transcriptional changes in the mantle tissue of *Ruditapes philippinarum* caused by Brown Ring Disease (a shell disease caused by the bacteria *Vibrio tapetis*) not only include genes related to biomineralization but also many immunity genes^24,25^ (such as big defensin, lectin, C1q domain containing protein and so on), implying the important role of mantle tissue in the immune response to pathogens.

Like all invertebrates, scallops rely on an exclusively innate immune system to execute cellular and humoural (macromolecules) immune reactions to resist invaders^26^. As the classical immune tissue in scallops, hemocytes use encapsulation and phagocytosis to eliminate undesirable particles (e.g., pathogens and abnormal/dead cells) performing the cellular immune reactions and contribute to the secretion of immune macromolecules to execute humoural immune responses, such as immune recognition, signal transduction and destruction of invaders, as reviewed by Song *et al*.^27^. Recent studies have also demonstrated a key role of hemocytes in biomineralization due to their involvement in mineral transport, as well as production of the extracellular matrix of the shells^28–31^, indicating the multifunction of hemocytes. Ivanina *et al*., recognized that potential trade-offs existed between biomineralization and immunity in *Crassostrea gigas* and *C. virginica*^29^, but relevant studies have not been reported in *M. yessoensis*.

In the present study, a comprehensive and niche-targeted cDNA sequences database was constructed for *M. yessoensis* infested by *Polydora*, transcriptional changes in the edge mantle, central mantle and hemocytes of the diseased scallops were first detected, and genes related to shell formation and immunity were screened. The aim was to reveal the molecular response mechanism of *M. yessoensis* to the infestation of *Polydora*, and provide more valuable information for the mechanism studies of biomineralization and immunomodulation.

## Results

### Transcriptome sequencing, assembly and functional annotation

To identify differently expressed genes between healthy and diseased *M. yessoensis* infested by *Polydora*, 18 cDNA libraries of the edge mantle, central mantle and hemocytes of two groups were separately constructed for RNA-seq sequencing. After removing adaptors and low-quality reads, a total of 942,738,120 reads (~109.7 Gb) with an average of 52,374,340 (~6.1 Gb) for each sample were obtained, and the detailed sequencing information for each library is listed in Supplementary Table S1. *De novo* assembly for the transcriptome sequences was chosen to construct a comprehensive and niche-targeted cDNA sequence database for diseased scallops, and the Trinity method was used with all trimmed reads. Finally, 80,831 unigenes were obtained with an average length of 1607 bp (Supplementary Fig. S1), and the assembly information is summarized in Supplementary Table S2. The unigenes were annotated by searching the sequences against the Nr, SWISS-PROT, KOG, GO and KEGG databases using BLASTX with a cut-off of Evalue ≤ 1e-5 to describe their functions at different levels, and the annotation ratios were 31.32%, 23.75%, 20.27%, 21.73% and 8.97%, respectively (Supplementary Table S3).

For GO classification, 17,567 unigenes were annotated into 12,658 GO terms in three main GO categories: biological process (~65.67%), cellular component (~10.44%), and molecular function (~23.90%) (Supplementary Table S4 and Fig. S2). In the biological process category, most of the unigenes were assigned to the terms of ‘cellular process’ (~14.81%), ‘single-organism process’ (~12.65%) and ‘metabolic process’ (~11.58%), including ‘immune system process’ (~1.15%). In the cellular component category, most of the unigenes were assigned to the terms of ‘cell’ (~19.54%), ‘cell part’ (~19.49%) and ‘organelle part’ (~15.29%). In the molecular function category, most of the unigenes were assigned to the terms of ‘binding’ (~44.48%), ‘catalytic activity’ (~33.17%) and ‘transporter activity’ (~5.95%). A total of 16,387 unigenes were assigned to 25 ortholog groups in the KOG database (Supplementary Table S5 and Fig. S3), which were distributed mainly into three groups: (R) ‘General function prediction only’ (~19.30%), (T) ‘Signal transduction mechanisms’ (~17.82%), and (O) ‘Posttranslational modification, protein turnover, chaperones’ (~10.02%). The biological signaling pathways for the unigenes were predicted by the KEGG database, and 7253 unigenes were assigned to 342 pathways (Table S6) involving six KEGG categories with ‘cellular processes’ (~11.81%), ‘environmental information processing’ (~13.76%), ‘genetic information processing’ (~7.56%), ‘human diseases’ (~25.05%), ‘metabolism’(~18.38%) and ‘organismal systems’ (~23.43%), in which the subcategories of ‘infectious diseases’, ‘immune diseases’, ‘nervous system’, ‘immune system’, etc. were contained (Supplementary Fig. S4).

### Overall gene regulation in diseased scallops

**Figure 2:**
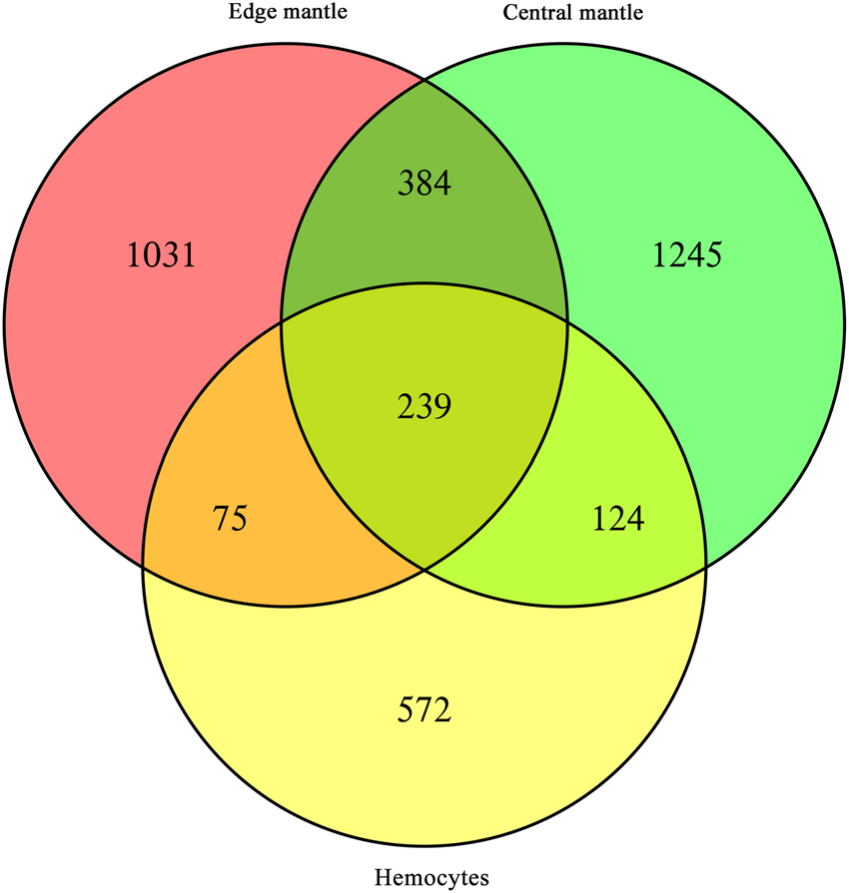
Distribution of differentially expressed unigenes between diseased and healthy *M. yessoensis* across different tissues (edge mantle, central mantle and hemocytes).

**Figure 3:**
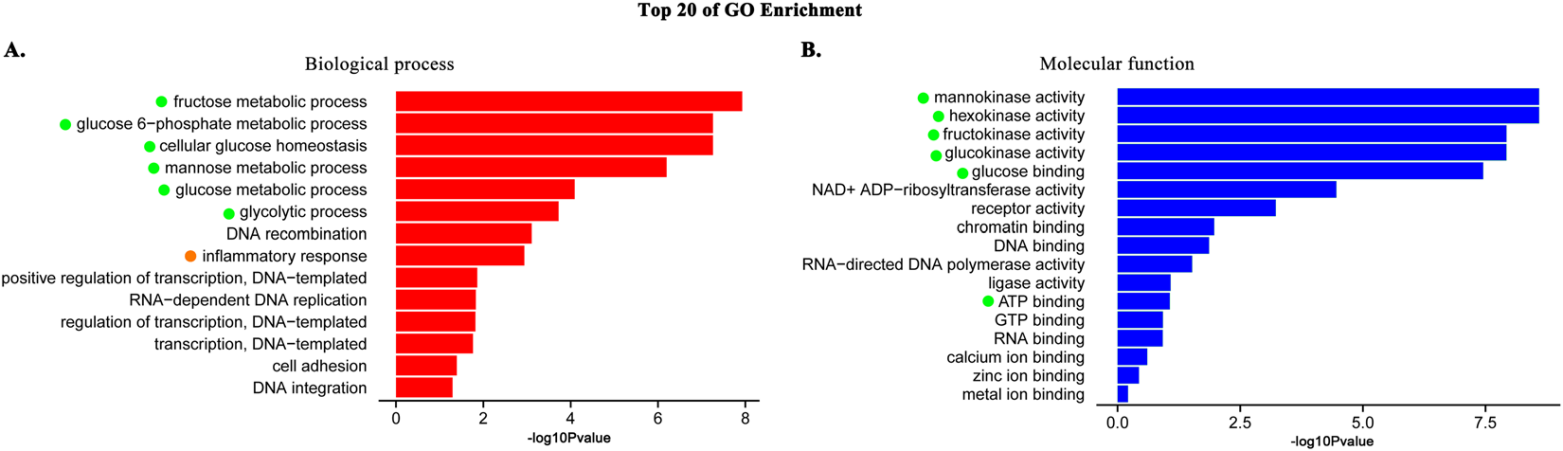
GO terms significantly enriched among DEGs shared by the edge mantle, central mantle and hemocytes. (**A**) Biological process. (**B**) Molecular function. Only the top 20 are presented, which are sorted by the p-value (p ≤ 0.05) and displayed with the –log_10_(p-value). Terms or pathways related to ‘glycometabolism’ and ‘immune response’ are separately highlighted with green and orange dots.

**Figure 4:**
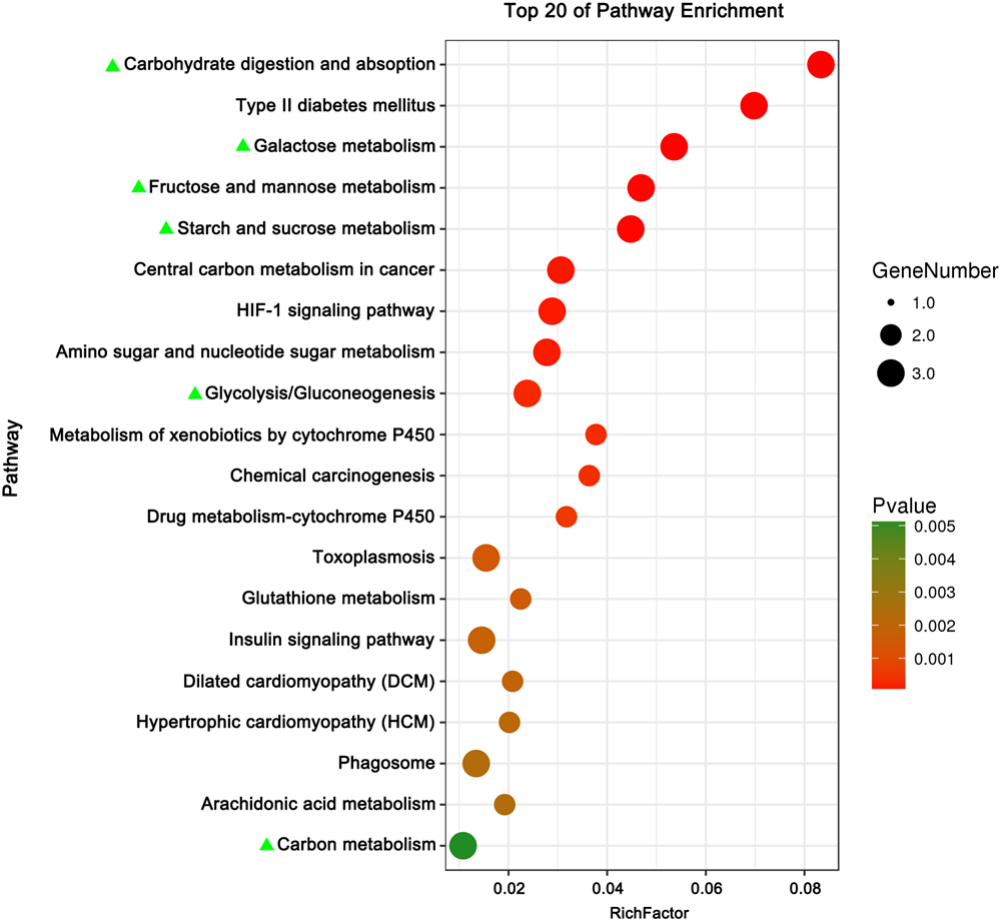
KEGG pathway enrichment analysis among DEGs shared by the three tissues. Only the top 20 are presented, which are sorted by the p-value (p ≤ 0.05). Terms or pathways related to ‘glycometabolism metabolism’ are highlighted with green triangles.

The expression levels of the assembled unigenes were estimated by the FPKM method. Gene differential expression analysis was conducted between healthy and infested scallops in the edge mantle, central mantle and hemocytes tissues (p ≤ 0.05). A total of 3670 unigenes were differentially expressed in at least one of the three tissues with 1729 in the edge mantle, 1992 in the central mantle and 1010 in the hemocytes. Among these DEGs, there were 822 DEGs (~22.4%) shared by at least two of the tissues with 623 between the edge and central mantles, 314 between the edge mantle and hemocytes and 363 between the central mantle and hemocytes. Moreover, 239 (~6.5%) DEGs were shared among all the three tissues (Fig. 2). It is noteworthy that these three-tissue-shared DEGs were significantly enriched in the functions and processes related to glycometabolism by GO and KEGG enrichment analysis (p ≤ 0.05) (Figs 3 and 4). For instance, the GO terms of the ‘fructose metabolic process’, ‘glucose 6−phosphate metabolic process’, ‘cellular glucose homeostasis’, ‘mannose metabolic process’, ‘glucose metabolic process’ and ‘glycolytic process’ were the most significantly enriched terms in the biological process category (Fig. 3A). Meanwhile, relevant molecular activity, such as ‘mannokinase activity’, ‘hexokinase activity’, ‘fructokinase activity’, ‘glucokinase activity’, ‘glucose binding’ and ‘ATP binding’ were also significantly overrepresented in the molecular function category (Fig. 3B). Additionally, KEGG enrichment analysis showed a similar trend with pathways related to glycometabolism being the most significantly enriched, including ‘carbohydrate digestion and absorption’, ‘galactose metabolism’, ‘fructose and mannose metabolism’, ‘starch and sucrose metabolism’, ‘glycolysis/gluconeogenesis’ and ‘carbon metabolism’ (Fig. 4). Moreover, all of the above relevant terms were found to be upregulated in all the three tissues (Tables 1–3), which likely implied very active energy metabolism in these tissues to meet the living demands of the diseased scallops.

**Table 1:** GO terms significantly enriched in the edge mantle.

iD	Term	p(≤0.05)	Type	iD	Term	p(≤0.05)	Type
**Upregulated in diseased scallops**				**Related to cellular motility**			
**Related to calcium ion**				GO:0005929	cilium	2.29E-12	C
GO:0060402	calcium ion transport into cytosol	6.53E-04	P	GO:0005874	microtubule	8.31E-11	C
GO:0005509	calcium ion binding	8.02E-04	F	GO:0031514	motile cilium	1.49E-10	C
GO:0051480	cytosolic calcium ion homeostasis	2.81E-03	P	GO:0003777	microtubule motor activity	2.89E-08	F
GO:0005262	calcium channel activity	5.00E-02	F	GO:0005930	axoneme	4.39E-08	C
**Related to shell organic matrix**				GO:0003341	cilium movement	8.41E-08	P
GO:0018392	glycoprotein 3-alpha-L-fucosyltransferase activity	1.88E-03	F	GO:1990716	axonemal central apparatus	1.08E-07	C
GO:0030203	glycosaminoglycan metabolic process	1.40E-02	P	GO:0035082	axoneme assembly	6.89E-07	P
**Related to nervous system**				GO:0030286	dynein complex	1.42E-06	C
GO:0021591	ventricular system development	4.77E-05	P	GO:0060294	cilium movement involved in cell motility	1.27E-05	P
GO:0021670	lateral ventricle development	1.15E-04	P	GO:0003351	epithelial cilium movement	3.22E-05	P
GO:0043083	synaptic cleft	4.64E-04	C	GO:0036158	outer dynein arm assembly	3.22E-05	P
GO:0007399	nervous system development	4.75E-04	P	GO:0036157	outer dynein arm	5.83E-05	C
GO:0010976	positive regulation of neuron projection development	2.96E-03	P	GO:0010632	regulation of epithelial cell migration	6.79E-05	P
GO:0007420	brain development	4.64E-03	P	GO:0007018	microtubule-based movement	9.43E-05	P
GO:0007417	central nervous system development	4.68E-03	P	GO:0060271	cilium morphogenesis	4.77E-04	P
GO:0006665	sphingolipid metabolic process	7.02E-03	P	GO:0036064	ciliary basal body	6.35E-04	C
GO:0045202	synapse	1.76E-02	C	GO:0042384	cilium assembly	1.09E-03	P
GO:0043005	neuron projection	3.21E-02	C	GO:0036156	inner dynein arm	1.50E-03	C
GO:0005328	neurotransmitter:sodium symporter activity	1.39E-02	F	GO:0001539	cilium or flagellum-dependent cell motility	2.32E-03	P
**Related to energy metabolism**				GO:0005858	axonemal dynein complex	2.81E-03	C
GO:0004550	nucleoside diphosphate kinase activity	3.94E-08	F	GO:0036159	inner dynein arm assembly	2.81E-03	P
GO:0006165	nucleoside diphosphate phosphorylation	3.62E-06	P	GO:0008017	microtubule binding	1.54E-02	F
GO:0009142	nucleoside triphosphate biosynthetic process	2.08E-05	P	GO:0072372	primary cilium	3.60E-02	C
GO:0016887	ATPase activity	2.58E-03	F	GO:0030334	regulation of cell migration	3.84E-02	P
GO:0004396	hexokinase activity	1.15E-04	F				
GO:0019158	mannokinase activity	1.15E-04	F	**Downregulated in diseased scallops**			
GO:0004340	glucokinase activity	3.13E-04	F	**Related to nervous system**			
GO:0006000	fructose metabolic process	3.13E-04	P	GO:0043523	regulation of neuron apoptotic process	2.40E-03	P
GO:0008865	fructokinase activity	3.13E-04	F	**Related to immume system**			
GO:0016051	carbohydrate biosynthetic process	5.06E-04	P	GO:0006954	inflammatory response	2.07E-03	P
GO:0005536	glucose binding	6.53E-04	F	GO:0051607	defense response to virus	6.20E-03	P
GO:0001678	cellular glucose homeostasis	8.86E-04	P	GO:0045087	innate immune response	1.00E-02	P
GO:0051156	glucose 6-phosphate metabolic process	8.86E-04	P	GO:0005044	scavenger receptor activity	1.31E-02	F
GO:0006013	mannose metabolic process	4.67E-03	P				
GO:0006006	glucose metabolic process	2.64E-02	P

**Table 2:** GO terms significantly enriched in the central mantle.

iD	Term	p(≤0.05)	Type	iD	Term	p(≤0.05)	Type
**Upregulated in diseased scallops**				**Related to nervous system**			
**Related to calcium ion**				GO:0007218	neuropeptide signaling pathway	2.18E-04	P
GO:0051480	cytosolic calcium ion homeostasis	1.28E-03	P	GO:0010976	positive regulation of neuron projection development	6.57E-03	P
GO:0046686	response to cadmium ion	4.63E-02	P	GO:0008188	neuropeptide receptor activity	7.47E-03	F
**Related to shell organic matrix**				GO:0006836	neurotransmitter transport	8.83E-03	P
GO:0006493				**Related to cell migration**			
GO:0018392	glycoprotein 3-alpha-L-fucosyltransferase activity	8.50E-04	F	GO:0010634	positive regulation of epithelial cell migration	2.92E-04	P
GO:0046920	alpha-(1->3)-fucosyltransferase activity	1.54E-03	F	GO:0061580	colon epithelial cell migration	5.24E-04	P
GO:0008417	fucosyltransferase activity	3.14E-03	F	GO:0010632	regulation of epithelial cell migration	6.75E-04	P
GO:0005578	proteinaceous extracellular matrix	1.17E-02	C				
GO:0005576	extracellular region	1.36E-02	C	**Downregulated in diseased scallops**			
GO:0006486	protein glycosylation	4.52E-02	P	**Related to shell organic matrix**			
GO:0070062	extracellular exosome	4.77E-02	C	GO:0005578	proteinaceous extracellular matrix	2.66E-03	C
**Related to energy metabolism**				GO:0030203	glycosaminoglycan metabolic process	3.05E-03	P
GO:0016160	amylase activity	0	F	GO:0005581	collagen trimer	2.84E-02	C
GO:0044245	polysaccharide digestion	0	P	GO:0005576	extracellular region	4.49E-02	C
GO:0005983	starch catabolic process	1.04E-05	P	**Related to nervous system**			
GO:0004396	hexokinase activity	5.07E-05	F	GO:0006687	glycosphingolipid metabolic process	1.95E-03	P
GO:0019158	mannokinase activity	5.07E-05	F	GO:0048813	dendrite morphogenesis	4.54E-03	P
GO:0004340	glucokinase activity	1.39E-04	F	GO:0001843	neural tube closure	1.23E-02	P
GO:0006000	fructose metabolic process	1.39E-04	P	GO:0045202	synapse	1.88E-02	C
GO:0008865	fructokinase activity	1.39E-04	F	**Related to immume system**			
GO:0005536	glucose binding	2.92E-04	F	GO:0034340	response to type I interferon	5.87E-04	P
GO:0001678	cellular glucose homeostasis	3.97E-04	P	GO:0032608	interferon-beta production	9.55E-04	P
GO:0051156	glucose 6-phosphate metabolic process	3.97E-04	P	GO:0006954	inflammatory response	1.96E-03	P
GO:0006013	mannose metabolic process	2.15E-03	P	GO:0051607	defense response to virus	4.21E-03	P
GO:0006006	glucose metabolic process	1.06E-02	P	GO:0005044	scavenger receptor activity	5.63E-03	F
GO:0005975	carbohydrate metabolic process	2.53E-02	P

**Table 3:** GO terms significantly enriched in the hemocytes.

iD	Term	p(≤0.05)	Type	iD	Term	p(≤0.05)	Type
**Upregulated in diseased scallops**				**Downregulated in diseased scallops**			
**Related to energy metabolism**				**Related to nervous system**			
GO:0004396	hexokinase activity	4.46E-06	F	GO:0007218	neuropeptide signaling pathway	5.24E-03	P
GO:0019158	mannokinase activity	4.46E-06	F	GO:0008188	neuropeptide receptor activity	9.90E-03	F
GO:0004340	glucokinase activity	1.24E-05	F	**Related to immume response**			
GO:0006000	fructose metabolic process	1.24E-05	P	GO:0002218	activation of innate immune response	3.53E-05	P
GO:0008865	fructokinase activity	1.24E-05	F	GO:0051607	defense response to virus	1.24E-03	P
GO:0005536	glucose binding	2.63E-05	F	GO:0002230	positive regulation of defense response to virus by host	1.75E-03	P
GO:0001678	cellular glucose homeostasis	3.60E-05	P	GO:0045087	innate immune response	1.52E-02	P
GO:0051156	glucose 6-phosphate metabolic process	3.60E-05	P	GO:0005044	scavenger receptor activity	1.67E-02	F
GO:0006013	mannose metabolic process	2.04E-04	P	GO:0006954	inflammatory response	1.70E-02	P
GO:0006006	glucose metabolic process	6.57E-03	P	GO:0006955	immune response	3.66E-02	P
GO:0006096	glycolytic process	1.19E-02	P				
GO:0016887	ATPase activity	1.67E-02	F				
GO:0005524	ATP binding	2.11E-02	F				
**Related to nervous system**							
GO:0005328	neurotransmitter:sodium symporter activity	1.23E-02	F				

### Gene regulation in different tissues

For the DEGs of the three tissues, there were 1162 (~67.21%) upregulated and 567 (~32.79%) downregulated in the edge mantle, 1040 (~52.21%) upregulated and 952 (~47.79%) downregulated in the central mantle, and 521 (~51.58%) upregulated and 489 (~48.42%) downregulated in hemocytes. GO enrichment analysis was separately carried out for the up and downregulated DEGs of each tissue. On the whole, a variety of functions were covered for the enriched GO terms, but many of them could be classified into one category according to their functions (Tables 1–3, Supplementary Fig. 5S), which were probably related to shell formation and immunomodulation, such as calcium ion binding and transport, shell organic matrix, nervous system development, epithelial cell motility and immune response, including the glycometabolism-related functions shared by the three tissues.

GO enrichment analysis for upregulated genes in the edge mantle are described in Table 1. First, some GO terms related to calcium ion activities, such as ‘calcium ion transport into cytosol’, ‘calcium ion binding’, ‘cytosolic calcium ion homeostasis’ and ‘calcium channel activity’ and shell organic matrix, such as ‘glycoprotein 3-alpha-L-fucosyltransferase activity’ and ‘glycosaminoglycan metabolic process’, were significantly enriched, which were closely related to the process of biomineralization. In addition, many GO terms related to cellular motility, especially the cilium movement of the epithelial cells, were also significantly enriched, and most of them ranked in the top 20 among all the enriched terms according to their significance level (Supplementary Fig. S5-A1, marked with red dots), such as the biological process for cilium assembly and movement with GO terms of ‘cilium movement’, ‘axoneme assembly’, ‘cilium movement involved in cell motility’, ‘outer dynein arm assembly’, ‘epithelial cilium movement’, ‘regulation of epithelial cell migration’, ‘microtubule-based movement’, the cellular component for the cilium with GO terms of ‘cilium’, ‘microtubule’, ‘motile cilium’, ‘axoneme’, ‘axonemal central apparatus’, ‘dynein complex’, ‘ciliary basal body’, and the molecular function for ‘microtubule motor activity’ and ‘microtubule binding’, suggesting exuberant activity of the epithelial cells. There was another notable enrichment related to the nervous system, including the biological process GO terms ‘ventricular system development’, ‘lateral ventricle development’, ‘nervous system development’, ‘positive regulation of neuron projection development’, ‘brain development’, ‘central nervous system development’, ‘sphingolipid metabolic process’ and ‘regulation of neuron apoptotic process’ (downregulated), cellular component GO terms ‘synaptic cleft’, ‘synapse’ and ‘neuron projection’, and molecular function GO term ‘neurotransmitter: sodium symporter activity’, implying the involvement of the nervous system in the modulation process after an infestation of *Polydora*. The final overrepresented GO terms were those related to energy metabolism and were similar to the other two tissues, including glycometabolism and ATP catabolism and anabolism related terms (e.g., ‘nucleoside diphosphate kinase activity’, ‘nucleoside diphosphate phosphorylation’, ‘nucleoside triphosphate biosynthetic process’ and ‘ATPase activity’). All the relevant GO terms are listed in Table 1, and those ranked in the top 20 are dotted with different colours in Supplementary Fig. 5S-A1.

In the central mantle, there was a similar distribution of the enriched GO terms for upregulated genes with terms related to calcium ion activity, shell organic matrix, energy metabolism, nervous system and cellular motility gathering, as well (Table 2; terms ranking in the top 20 are dotted with different colours in Supplementary Fig. 5S-A2). However, differences still existed between the two regions. For example, more terms related to the shell organic matrix were enriched in the central mantle and some showed upregulation (Cellular component: ‘proteinaceous extracellular matrix’, ‘extracellular region’ and ‘extracellular exosome’. Biological process: ‘protein O-linked glycosylation’ and ‘protein glycosylation’. Molecular function: ‘glycoprotein 3-alpha-L-fucosyltransferase activity’, ‘alpha-(1- > 3)-fucosyltransferase activity’, ‘fucosyltransferase activity’), while some also showed downregulation (Cellular component: ‘proteinaceous extracellular matrix’, ‘collagen trimer’ and ‘extracellular region’. Biological process: ‘glycosaminoglycan metabolic process’). For the terms related to the nervous system, fewer showed upregulation (‘neuropeptide signaling pathway’, ‘positive regulation of neuron projection development’, ‘neurotransmitter transport’ and ‘neuropeptide receptor activity’), but more showed downregulation (‘synapse’, ‘glycosphingolipid metabolic process’, ‘dendrite morphogenesis’ and ‘neural tube closure’). GO terms related to cellular motility were mainly focused on epithelial cell migration (‘positive regulation of epithelial cell migration’, ‘colon epithelial cell migration’ and ‘regulation of epithelial cell migration’), and terms related to cilium movement were not found in the central mantle. Different regulation processes probably occurred in the two mantle regions suggesting their different tissue functions in shell formation.

Among the downregulated DEGs, many immune-related GO terms were detected in all three tissues, especially in hemocytes (Tables 1–3), and most of them ranked in the top 20 among all the enriched terms (Supplementary Fig. 5S-B dotted with orange colour). For example, four related GO terms (‘inflammatory response’, ‘defense response to virus’, ‘innate immune response’ and ‘scavenger receptor activity’) were enriched in the edge mantle, and five (‘response to type I interferon’, ‘interferon-beta production’, ‘inflammatory response’, ‘defense response to virus’ and ‘scavenger receptor activity’) in the central mantle, while seven were enriched in hemocytes (‘activation of innate immune response’, ‘defense response to virus’, ‘positive regulation of defense response to virus by host’, ‘innate immune response’, ‘inflammatory response’, ‘immune response’ and ‘scavenger receptor activity’). The term ‘inflammatory response’ was also enriched for the three-tissue-shared DEGs (Fig. 3A, dotted with orange colour). However, no upregulated genes related to immunity were found to be significantly enriched, probably indicating the declining immune competence in diseased scallops. In addition, nervous system-related GO terms were also found enriched in hemocytes, with ‘neurotransmitter: sodium symporter activity’ being upregulated similarly with the edge and central mantle, while ‘neuropeptide signaling pathway’ and ‘neuropeptide receptor activity’ were downregulated, indicating the neuromodulation in the immune response of *M. yessoensis*.

### Genes related to biomineralization, immune and nervous system

Many biomineralization-related genes were differentially expressed in mantle tissues (Table 4), and most of them showed upregulation, which suggested a more active shell formation in the infested scallops. The functions of these genes were mainly associated with calcium binding or transport, such as ‘calbindin’ (CL3204Contig1), ‘calmodulin (CL51277Contig1), ‘EF-hand calcium-binding domain-containing protein’ (CL3228Contig1, CL3161Contig1, CL8277Contig1), ‘sodium/calcium exchanger’ (CL36832Contig1) and ‘transient receptor potential cation channel’ (CL2149Contig2, CL1979Contig2, CL1618Contig1) and shell matrix proteins, such as ‘collagen’ (CL12529Contig1, CL2550Contig1), ‘laminin’ (CL27Contig2), ‘von Willebrand factor A domain-containing protein’ (CL41489Contig1, CL721Contig1), ‘sclerostin’ (CL12811Contig1), ‘glycoprotein’ (CL55247Contig1,CL4117Contig1), ‘fibronectin ‘(CL15874Contig1), ‘glutathione peroxidase’ (CL29217Contig1), ‘hephaestin’ (CL12821Contig1), ‘ferritin’ (comp87468_c1_seq2_1), ‘tenascin’ (CL27Contig7, CL1236Contig1), ‘chitotriosidase ‘(CL50207Contig1), ‘laccase’ (CL6551Contig1), ‘tyrosinase-like proteins’ (CL14061Contig1) and ‘carbohydrate sulfotransferase’ (CL6600Contig1, CL2673Contig1, CL55429Contig1). Most of these genes were modulated in only one region of the mantle, implying different gene activation in two regions to form the different shell layers.

**Table 4:** Genes related to biomineralization in the mantle tissue.

Transcript	Tissue	Fold	p(≤0.05)	Regulated	Description	e-value
**Calcium binding or transport**						
CL3204Contig1	edge mantle	3.23	1.01E-02	up	Calbindin-32	2.00E-83
CL3204Contig1	central mantle	4.02	1.13E-04	up	Calbindin-32	2.00E-83
CL51277Contig1	edge mantle	5.04	4.41E-02	up	Calmodulin	7.00E-34
CL36832Contig1	edge mantle	4.78	1.55E-02	up	Sodium/calcium exchanger 3	3.00E-168
CL3228Contig1	edge mantle	5.40	2.47E-02	up	EF-hand calcium-binding domain-containing protein 1	3.00E-41
CL3161Contig1	edge mantle	3.13	3.87E-02	up	EF-hand calcium-binding domain-containing protein 5	2.00E-154
CL8277Contig1	edge mantle	3.71	2.16E-02	up	EF-hand calcium-binding domain-containing protein 6	4.00E-175
CL2149Contig2	edge mantle	3.84	1.56E-02	up	Transient receptor potential cation channel trpm	3.00E-168
CL1979Contig2	central mantle	8.45	8.21E-03	up	Transient receptor potential cation channel subfamily M member 6	8.00E-62
CL1618Contig1	central mantle	3.95	1.58E-02	up	Short transient receptor potential channel 3	1.00E-167
**Shell matrix protein**						
CL12529Contig1	edge mantle	25.04	6.07E-03	up	Collagen alpha-5(VI) chain	7.00E-60
CL2550Contig1	central mantle	3.13	1.70E-02	up	Collagen alpha-1(XII) chain	1.00E-25
CL27Contig2	edge mantle	4.65	4.53E-03	up	Laminin subunit alpha-5	7.00E-08
CL721Contig1	edge mantle	3.28	1.49E-02	up	Von Willebrand factor A domain-containing protein 3A	0
CL41489Contig1	edge mantle	4.86	1.03E-02	up	Von Willebrand factor A domain-containing protein 3B	1.00E-18
CL12821Contig1	central mantle	6.55	1.72E-04	up	Hephaestin-like protein	0
CL27Contig7	edge mantle	Inf	6.25E-03	up	Tenascin	8.00E-27
CL1236Contig1	central mantle	3.26	2.80E-02	up	Tenascin-X	1.00E-32
CL55247Contig1	edge mantle	Inf	4.52E-02	up	Glycoprotein 3-alpha-L-fucosyltransferase A	9.00E-45
CL55247Contig1	central mantle	97.22	3.36E-04	up	Glycoprotein 3-alpha-L-fucosyltransferase A	9.00E-45
CL4117Contig1	central mantle	9.39	4.80E-03	up	Glycoprotein-N-acetylgalactosamine 3-beta-galactosyltransferase 1	7.00E-92
CL6551Contig1	edge mantle	3.33	3.81E-02	up	Laccase-2	8.00E-53
CL6551Contig1	central mantle	2.61	2.48E-02	up	Laccase-2	8.00E-53
CL14061Contig1	central mantle	2.30	1.79E-02	up	Putative tyrosinase-like protein tyr-3	3.00E-38
CL6600Contig1	edge mantle	6.80	2.24E-02	up	Carbohydrate sulfotransferase 8	5.00E-21
CL55429Contig1	edge mantle	9.51	2.12E-02	up	Carbohydrate sulfotransferase 11	2.00E-27
CL2673Contig1	central mantle	−3.53	4.55E-02	down	Carbohydrate sulfotransferase 9	8.00E-29
CL12811Contig1	central mantle	−3.86	2.44E-03	down	Sclerostin domain-containing protein 1	1.00E-17
CL15874Contig1	edge mantle	−2.92	2.73E-02	down	Fibronectin-like	1.69E-06
CL29217Contig1	edge mantle	−4.09	4.23E-02	down	Glutathione peroxidase	2.00E-68
comp87468_c1_seq2_1	central mantle	−2.25	2.33E-02	down	Soma ferritin	1.00E-50
CL50207Contig1	central mantle	−13.44	2.09E-02	down	Chitotriosidase-1	3.96E-08

Many immune-related genes were also modulated in the two mantle regions and hemoctyes of the infested scallops (Table 5). The functions of these genes could be classified into the following categories: I) Immune recognition, e.g., ‘lectin’ (CL33366Contig1), ‘toll-like receptor (TLR)’ (CL12334Contig1, comp147899_c0_seq5_3), ‘scavenger receptor’ (CL63572Contig1), ‘C1q domain containing proteins’ (CL50918Contig1) and ‘fibrinogen-related proteins’ (CL26062Contig1, comp144120_c0_seq2_3). II) Immune effectors, e.g., ‘g-type lysozyme’ (CL3126Contig2), ‘superoxide dismutase (SOD)’ (comp96557_c0_seq1_1, comp119797_c0_seq1_3), ‘catalase’ (CL10534Contig1), ‘big defensin’ (CL8776Contig1) and ‘heat shock proteins (HSPs)’ (CL1392Contig1, CL37312Contig1, CL1984Contig1, CL47124Contig1, CL2340Contig1). III) Signal transduction, e.g., ‘tumor necrosis factor (TNF) receptor’ (CL23037Contig1, comp110989_c0_seq6_2), ‘serine protease’ (CL61221Contig1) and ‘NF-kappa-B inhibitor’ (comp122698_c0_seq6_2). Other genes were included, such as ‘immunoglobulin’ (CL47987Contig1, CL19464Contig1), ‘integrin’ (comp127636_c0_seq5_2), ‘interleukin’ (CL13815Contig1), ‘interleukin receptor’ (CL13604Contig1), ‘stimulator of interferon genes protein’ (comp129146_c0_seq2_3) and ‘pathogen-related protein’ (comp52531_c0_seq2_1). Unexpectedly, many of these genes showed downregulation.

**Table 5:** Genes related to the immune response in the mantle tissue and hemocytes.

Transcript	Tissue	Fold	p(≤0.05)	Regulated	Description	e-value
**Immune recognition**						
CL33366Contig1	hemocytes	10.89	7.19E-04	up	Lectin	2.00E-50
CL33366Contig1	edge mantle	20.09	1.63E-03	up	Lectin	2.00E-50
CL33366Contig1	central mantle	8.76	5.93E-09	up	Lectin	2.00E-50
CL26062Contig1	edge mantle	3.58	2.28E-02	up	Fibrinogen C domain-containing protein 1	1.00E-67
comp144120_c0_seq2_3	central mantle	−5.41	3.75E-02	down	Fibrinogen C domain-containing protein 1	3.00E-52
CL12334Contig1	hemocytes	−46.78	2.41E-03	down	Toll-like receptor 8	7.00E-19
CL12334Contig1	edge mantle	−36.55	3.47E-03	down	Toll-like receptor 8	7.00E-19
CL12334Contig1	central mantle	−17.80	1.22E-02	down	Toll-like receptor 8	7.00E-19
comp147899_c0_seq5_3	hemocytes	−7.31	2.66E-02	down	Toll-like receptor 1	2.00E-29
comp147899_c0_seq5_3	edge mantle	−8.58	3.53E-05	down	Toll-like receptor 1	2.00E-29
comp147899_c0_seq5_3	central mantle	−5.32	5.59E-05	down	Toll-like receptor 1	2.00E-29
CL63572Contig1	hemocytes	−3.19	2.13E-02	down	Scavenger receptor class F member 1	2.00E-13
CL50918Contig1	hemocytes	−10.76	3.59E-02	down	Complement C1q tumor necrosis factor-related protein 2	4.00E-12
**Immune effectors**						
CL3126Contig2	hemocytes	9.86	3.66E-02	up	G-type lysozyme	7.44E-138
comp96557_c0_seq1_1	hemocytes	Inf	7.36E-03	up	Copper/zinc superoxide dismutase	1.33E-07
comp119797_c0_seq1_3	edge mantle	383.06	3.75E-06	up	Copper/zinc superoxide dismutase	2.02E-07
CL10534Contig1	central mantle	2.31	1.68E-02	up	Catalase	0
CL8776Contig1	central mantle	4.04	3.27E-03	up	Big defensin	1.00E-35
CL1392Contig1	hemocytes	9.06	4.25E-02	up	Heat shock protein STI1	1.00E-09
CL47124Contig1	central mantle	4.07	9.89E-03	up	Heat shock 70kDa protein 12A	4.00E-59
CL37312Contig1	hemocytes	−124.11	1.06E-03	down	Heat shock 70kDa protein 12B	9.00E-92
CL2340Contig1	central mantle	−2.67	1.18E-02	down	Heat shock 70kDa protein 12B	3.00E-45
CL1984Contig1	edge mantle	−17.45	3.67E-02	down	Heat shock protein 90	0
**Signal transduction**,						
CL61221Contig1	central mantle	3.04	3.40E-02	up	Serine protease 23	1.00E-09
comp122698_c0_seq6_2	edge mantle	2.76	3.88E-02	up	NF-kappa-B inhibitor cactus	1.00E-36
comp110989_c0_seq6_2	central mantle	10.17	1.38E-02	up	Tumor necrosis factor receptor superfamily member 11B	8.00E-18
CL23037Contig1	hemocytes	−5.71	1.09E-02	down	Tumor necrosis factor receptor superfamily member 19L	1.00E-05
**Other related genes**						
CL47987Contig1	hemocytes	82.24	2.77E-02	up	Immunoglobulin superfamily member 10	9.00E-09
CL47987Contig1	central mantle	65.51	1.08E-03	up	Immunoglobulin superfamily member 10	9.00E-09
comp129146_c0_seq2_3	central mantle	5.04	2.10E-02	up	Stimulator of interferon genes protein	5.00E-15
comp52531_c0_seq2_1	central mantle	32.68	8.65E-04	up	Pathogen-related protein	1.00E-33
CL13604Contig1	edge mantle	3.49	4.51E-02	up	Interleukin-6 receptor subunit beta	1.00E-06
CL13815Contig1	edge mantle	−7.68	1.13E-02	down	Interleukin 17-like protein	2.00E-06
CL19464Contig1	hemocytes	Inf	1.84E-02	down	Low affinity immunoglobulin epsilon Fc receptor	3.00E-21
comp127636_c0_seq5_2	hemocytes	−5.26	4.50E-02	down	Integrin beta-6	3.00E-114
comp127636_c0_seq5_2	edge mantle	−17.01	4.63E-02	down	Integrin beta-6	3.00E-114
comp127636_c0_seq5_2	central mantle	−7.29	2.75E-02	down	Integrin beta-6	3.00E-114

In addition, some neural genes were differentially expressed in the three tissues (Table 6). Most of the genes showed upregulation, especially for genes for Na^+^/Cl^−^ dependent neurotransmitter transporters, such as the ‘GABA transporter’(comp139403_c0_seq2_3), ‘glycine transporter’ (CL50297Contig1), ‘aurine transporter’(CL31867Contig1), ‘proline transporter’ (CL51047Contig1) and ‘creatine transporter’ (CL11023Contig1)in the edge mantle, ‘GABA transporter’ (CL6833Contig1) in the central mantle, and ‘glycine transporter’ (CL22349Contig1) in hemocytes. However, the gene for ‘neurotrypsin’ (CL8026Contig1) was downregulated in all the three tissues. Nevertheless, genes for some neurotransmitter receptors had different situations. For example, the gene for ‘neuronal acetylcholine (ACh) receptor’ (CL31305Contig1) was up modulated in the edge mantle, while downregulated in the central mantle (CL42803Contig1) and hemocytes (CL35487Contig1). In contrast to the ACh receptor, the gene for the ‘5-hydroxytryptamine (5-HT) receptor’ was upregulated in both the central mantle and hemocytes but was not detected in the edge mantle. Some neuropeptide and neuropeptide receptor genes, such as the ‘myomodulin neuropeptides’ (CL32152Contig1) and ‘neuropeptide S receptor’ (CL939Contig1), ‘FMRF-amide neuropeptides’ (CL65515Contig1) and ‘FMRF-amide receptor’ (comp119939_c1_seq1_2) showed upregulation in the central mantle, while ‘FMRF-amide receptor’ (CL24831Contig) was downregulated in the hemocytes. There were also other neural-related genes were modulated in the three tissues as listed in Table 6. Genes found in this part (Table 6) probably mediated the processes of shell repair or immune response through neuromodulation.

**Table 6:** Genes related to the nervous system in the mantle tissue and hemocytes.

Transcript	Tissue	Fold	p(≤0.05)	Regulated	Description	e-value
CL11023Contig1	edge mantle	6.62	1.68E-02	up	Creatine transporter	2.00E-117
CL51047Contig1	edge mantle	3.63	2.72E-02	up	Proline-rich transmembrane protein 1	3.00E-07
comp139403_c0_seq2_3	edge mantle	3.08	1.97E-02	up	Sodium- and chloride-dependent GABA transporter 3	2.00E-75
CL50297Contig1	edge mantle	Inf	2.40E-02	up	Sodium- and chloride-dependent glycine transporter 1	1.00E-123
CL31867Contig1	edge mantle	4.80	2.67E-02	up	Sodium- and chloride-dependent taurine transporter	1.00E-18
CL6833Contig1	central mantle	2.32	4.72E-02	up	Sodium- and chloride-dependent GABA transporter 2	2.00E-140
CL41638Contig1	central mantle	2.67	1.12E-02	up	Synaptic vesicular amine transporter	2.00E-170
CL29255Contig1	hemocytes	57.78	1.00E-02	up	Sodium-dependent neutral amino acid transporter B(0)AT2	4.00E-168
CL22349Contig1	hemocytes	82.69	2.19E-02	up	Sodium- and chloride-dependent glycine transporter 2	4.00E-99
CL32152Contig1	central mantle	4.04	2.65E-02	up	Myomodulin neuropeptides 2	9.00E-09
CL939Contig1	central mantle	3.83	1.04E-03	up	Neuropeptide S receptor	6.00E-06
CL65515Contig1	central mantle	2.36	2.48E-02	up	FMRF-amide neuropeptides	2.00E-29
comp119939_c1_seq1_2	central mantle	3.59	4.65E-04	up	FMRFamide receptor	2.00E-19
CL24831Contig1	hemocytes	−2.67	2.33E-02	down	FMRFamide receptor	6.00E-19
CL31305Contig1	edge mantle	2.84	3.59E-02	up	Neuronal acetylcholine receptor subunit alpha-6	4.00E-81
CL42803Contig1	central mantle	−2.40	3.32E-02	down	Neuronal acetylcholine receptor subunit alpha-6	3.00E-33
CL35487Contig1	hemocytes	−3.64	3.71E-02	down	Neuronal acetylcholine receptor subunit alpha-10	2.00E-90
CL32064Contig1	central mantle	2.49	3.80E-02	up	5-hydroxytryptamine receptor 1	5.00E-07
CL1569Contig2	central mantle	2.26	4.96E-02	up	5-hydroxytryptamine receptor	2.00E-96
CL1569Contig2	hemocytes	15.57	3.47E-02	up	5-hydroxytryptamine receptor	2.00E-96
CL8026Contig1	edge mantle	−11.23	3.77E-02	down	Neurotrypsin	2.00E-12
CL8026Contig1	central mantle	−66.03	3.35E-03	down	Neurotrypsin	2.00E-12
CL8026Contig1	hemocytes	−2.43	4.80E-02	down	Neurotrypsin	2.00E-12
CL50512Contig1	edge mantle	2.55	3.87E-02	up	Advillin	1.00E-82
CL2411Contig2	edge mantle	38.46	3.02E-02	up	Ninjurin-1	2.00E-08
CL2411Contig1	hemocytes	−10.71	4.72E-02	down	Ninjurin-1	7.00E-12
CL3371Contig1	central mantle	−2.53	1.54E-02	down	Agrin	6.00E-120
CL6573Contig1	hemocytes	7.51	4.77E-03	up	Leukocyte tyrosine kinase receptor	2.00E-80
CL30510Contig1	hemocytes	−3.22	3.07E-02	down	Orexin receptor type 2	3.00E-07

## Discussion

The main objective of this study was to identify molecular changes in *M. yessoensis* caused by the infection of *Polydora* and explore the molecular response mechanism of the scallops. The study mainly focused on the transcriptional changes in the tissues of edge mantle (producing the periostracal and prismatic layers of the shell), central mantle (producing the nacreous layer of the shell) and hemocytes (classic immune tissue in scallops), which were directly related to the disease. The results showed significant alterations associated with the processes of biomineralization and immunomodulation in scallops to resist infection.

Changes related to biomineralization mainly occurred in the edge and central mantles. Genes related to calcium binding, calcium transporting and shell matrix proteins were significantly differentially expressed between healthy and diseased scallops (Table 4), and the relevant gene functions were also significantly enriched in the GO enrichment analysis (Tables 1 and 2). Calcium carbonate (95–99%) and shell matrix proteins (1–5%) are the main components of the shell^32,33^. Genes related to calcium binding and transporting regulate calcium concentration in the shell matrix and initiate calcium carbonate deposition, while the nucleation, growth and spatial orientation of calcium carbonate crystals are regulated by shell matrix proteins^34–39^. Notably, most of the involved genes, such as genes for calbindin, calmodulin, EF-hand calcium-binding domain-containing protein, collagen, laminin, tenascin, laccase, tyrosinase-like protein, carbohydrate sulfotransferase and others showed obvious upregulation in the present study, suggesting increased shell secretion activity in infested scallops to repair the damaged shells. This hypothesis was also proved by the extremely active epithelial cells, which secrete the organic matrix involved in shell formation^40^. As the results in Tables 1, 2 and Supplementary Fig. S5-A1 show, functions related to the activities of mantle epithelial cells were significantly upregulated and enriched, especially for those related to cilium motility, probably to accelerate the secretion and transport of matrix proteins needed for shell repair.

Differences existed between the two mantle regions with many genes regulated in only one region, indicating the different molecular functions of the two regions. For instance, genes for the calmodulin, sodium/calcium exchanger, EF-hand calcium-binding domain-containing protein, laminin, von Willebrand factor A domain-containing protein, fibronectin, glutathione peroxidase, etc. were only found to be differentially expressed in the edge mantle, while genes for the short transient receptor potential channel, hephaestin-like protein, tyrosinase-like protein, sclerostin domain-containing protein, ferritin, chitotriosidase, etc. were only in the central mantle, implying these genes possibly participate in different shell layers formation. In addition, some new shell matrix protein genes were first found in scallops in this study, i.e., genes for glutathione peroxidise and hephaestin, which were once thought to be nonfunctional or lost in the mollusk shell and independently arose in *Lingula* and corals^41^, but possibly function in scallops shell repair. Therefore, this work provides significant candidate genes for the mechanism study of shell formation, including genes involved in the different shell layers formation, and more research is needed to verify how these genes function.

Although hemocytes have been proved playing an important role in biomineralization in oyster due to their involvement in mineral transport as well as the production of the extracellular matrix of the shells^28–31^, the present study didn’t show obvious changes related to biomineralization in the hemocytes by gene differential expression analysis and GO and KEGG enrichment analysis. However, many genes related to immunomodulation were significantly differentially expressed in hemocytes and mantle tissue (Table 5) and the relevant GO terms were also significantly enriched (Tables 1–3, Supplementary Fig. 5SB). The results suggest that hemocytes may mainly play an immune function during *Polydora* infection in scallops and the mantle tissue was simultaneously involved in this process. As an invertebrate, scallops lack adaptive immunity and have evolved a series of sophisticated strategies to recognize and eliminate various invaders by employing a set of molecules to participate in immune recognition (e.g., lectins^42^, galectins^43,44^ and scavenge receptors^45^), signal transduction (e.g., serine protease cascades^46–48^, TLR^49,50^ and TNF^51^ signalling pathways) and incapacitation and elimination of invaders (e.g., lysozymes^52^, heat shock protein^53,54^, and antimicrobial peptides^55,56^). Immune-related genes detected in the present study (Table 5) are involved in all the processes mentioned above, which reflected an integrated immune response reaction in the scallops for the first time. Unexpectedly, a number of these genes showed significant downregulation, such as genes for toll-like receptor, scavenger receptor, complement C1q tumor necrosis factor-related protein, heat shock protein 70 and 90, etc. Furthermore, immune-related GO functions were significantly enriched in the downregulated but not upregulated genes, such as ‘inflammatory response’, ‘defense response to virus’, ‘innate immune response’, ‘scavenger receptor activity’, ‘activation of innate immune response’, etc., which indicated declining immunity of the infested scallops. Potential trade-offs between biomineralization and immunity has been demonstrated in *C. gigas* and *C. virginica*^29^. The expression of biomineralization-related genes was higher in *C. virginica* than in *C. gigas*, while expression was lower for immunity genes. Competition existed between these two processes. The contrasting regulation of biomineralization (upregulated on the whole) and immunity (downregulated) genes in the present study agreed with the above findings.

In addition, the nervous system likely played an important role in the response process. A number of neural genes were significantly differentially expressed in the two mantle regions and hemocytes (Table 6) and relevant GO functions were significantly enriched, as well (Tables 1–3, Supplementary Fig. 5S), suggesting the involvement of neuromodulation in the response process. Recently, neural-immune regulation in hemocytes was identified in mollusks; this regulation was conducted by neurotransmitters via a “nervous-hemocyte”-mediated neuroendocrine immunomodulatory axis (NIA)-like pathway^57,58^. In the present study, GO terms associated with neurotransmitter activity were significantly enriched in the mantle and hemocytes. Though genes for neurotransmitter were not detected among DEGs, genes for several neurotransmitter transporters and receptors were significantly differently expressed, such as Na^+^/Cl^−^ dependent neurotransmitter transporters, ACh receptor and 5-HT receptor, which partly implied the existence of neural immunomodulation in *M. yessoensis*. Few studies about neuromodulation in shell formation have been reported. The neural-associated DEGs in the mantle tissues could not be excluded from the regulation process of shell formation in this study, which offers a new perspective on the mechanism of shell formation; however, further exploration is needed.

Finally, scallops likely needed more energy during the response process. As we know, glycometabolism is the main pathway to supply energy for the organism. The results showed that many glycometabolism-related GO terms and pathways, e.g., glucose metabolic, fructose metabolic and mannose metabolic, were significantly enriched in the upregulated genes among all three tissues (Tables 1–3, Figs 3, 4 and Supplementary Fig. 5SA), which indicated an exuberant energy metabolism process in the diseased scallops. The generated energy was probably used for shell repair, immune reactions or other activities. For example, the active activity of epithelial cells in mantle tissues inevitably consumed a lot of energy. Consequently, more energy was used for survival and less energy remained for growth.

## Conclusions

To investigate the molecular response mechanisms of *M. yessoensis* infested by *Polydora*, a comprehensive and niche-targeted cDNA sequences database for diseased scallops was constructed and transcriptional changes in the edge mantle, central mantle and hemocytes were first detected in this study. The gene differential expression and enrichment analysis showed that the infestation of *Polydora* caused expression changes for genes or pathways involved in shell formation and immune response. Different mantle regions had different molecular functions probably responsible for the formation of different shell layers. An intact immune response process from immune recognition to pathogen elimination was first detected in scallops. Finally, neuromodulation and glycometabolism were involved in the infection response. These results provide a better understanding of shell formation and innate immune response in scallops and provide valuable resources for the genetic selection of disease-resistant scallops.

## Materials and Methods

### Sample collection and RNA extraction

Two-year-old healthy and infested *M. yessoensis* (Fig. 1) were collected from the Dalian Zhangzidao Sea. The two groups of scallops were acclimated in the laboratory for one week prior to the experiments. Filtered and aerated seawater was maintained at approximately 8 °C, which is within the optimum temperature range for their growth. Three scallops as biological replicates from each group were sampled, and tissues of the edge mantle, central mantle and hemocytes that had a direct correlation with the disease were immediately placed in liquid nitrogen and stored at −80 °C. Total RNA was isolated from each sample using an RNAprep pure tissue kit (Tiangent, China). The quantity and quality of total RNA were determined using the NanoDrop2000 spectrophotometer (Thermo Scientific, Wilmington, DE, USA) and agarose gel electrophoresis.

### cDNA library constructionand Illumina sequencing

mRNA was purified from total RNA by oligo(dT) magnetic beads, and each paired-end cDNA library was generated using the TruSeq RNA Sample Preparation Kit (Illumina, Inc., USA), following the manufacturer’s protocol. The quality of the libraries was assessed using the Agilent 2100 Bioanalyzer (Agilent Technologies, USA). Finally, a total of 18 cDNA libraries were sequenced on the Illumina HiSeq2500 genomic sequencing platform.

### Sequencing data processing, assembly and annotation

Raw sequencing data was processed to remove reads containing adapters or ambiguous “N” nucleotides (length exceeded 35 bp) and low quality reads (length of the bases with a quality score less than 20 exceeded 30%). The high-quality clean reads were combined and used for transcriptome assembly by the Trinity software (version: trinityrnaseq_r20131110)^59^ with default parameters. The generated contigs were clustered into unigenes by the TGICL software^60^, which were used as the reference sequences for subsequent analyses. The raw data have been deposited into the NCBI SRA database (accession number: SRP150161).

All the unigene sequences were aligned to the NCBI non-redundant protein (Nr) database, Swiss-Prot database, the Eukaryotic Orthologous Groups (KOG) protein database, Gene Ontology (GO) database and the Kyoto Encyclopedia of Genes and Genomes (KEGG) pathway database by BLASTX with an E-value of E ≤ 1e-5 to obtain the best putative functional annotations of each unigene.

### Gene differential expression analysis

The expression levels of the unigenes were determined by mapping the reads from the samples to reference unigenes using Bowtie 2 software (http://bowtie-bio.sourceforge.net)^61^. The FPKM (fragments per kilobase per million mapped reads) method^62^ was used to calculate the expression levels of the unigenes for each sample, which eliminated the effects of gene length and sequencing depth on the calculation of gene expression. Significance tests for expression differences in the mantle and hemocytes between healthy and infested scallops were conducted with negative binomial distribution hypothesis-testing, and the false discovery rate (FDR) method was applied to multiple tested hypotheses to correct the significant levels (p-values) and eliminate the influence of random fluctuations and errors. After calibration, a fold change of 2-fold and p ≤ 0.05 was set as the threshold. Enrichment analyses were performed by mapping all DEGs (differentially expressed genes) to GO and KEGG databases. GO functions or pathways with the statistical significance (p-value) lower than 0.05, which were evaluated by the hypergeometric distribution test, were considered to be enriched. The DEGs, enriched GO terms and KEGG pathways related to shell formation and immunity were highlighted.

## Electronic supplementary material

Supplementary Tables and Figures

Dataset 1
