# Supplementary material for: Transcriptional changes in the Japanese scallop (Mizuhopecten yessoensis) shellinfested by Polydora provide insights into the molecular mechanism of shell formation and immunomodulation
Source: Sci Rep. 2018 Dec 5;8:17664. doi: 10.1038/s41598-018-35749-x (PMC6281612; doi:10.1038/s41598-018-35749-x)
Supplement: Supplementary file 1 — Supplementary Tables and Figures [file 41598_2018_35749_MOESM1_ESM.pdf]

Transcriptional changes in the Japanese scallop (*Mizuhopecten yessoensis*) shellinfested by *Polydora* provide insights into the molecular mechanism of shell formation and immunomodulation

Junxia Mao[1], Wenjing Zhang[1], Xiaosen Zhang[1], Ying Tian[1], Xubo Wang[1], Zhenlin Hao[1], Yaqing Chang[1,*]

Supplementary Tables

Table S1: Transcriptome sequencing for different tissues of healthy and infested *M. yessoensis*.

Tissue	Sample	Clean reads	Clean bases
**Edge mantle**	Ed_infested_s1	52,298,880	6,536,565,266
	Ed _infested_s2	52,488,740	6,560,299,149
	Ed _infested_s3	52,370,900	6,545,565,850
	Ed _healthy_s1	52,219,740	6,526,671,504
	Ed _healthy_s2	52,317,560	6,538,901,786
	Ed _healthy_s3	52,248,200	6,530,237,932
	**Sum**	**313,944,020**	**39,238,241,487**
**Central mantle**	Ce_infested_s1	52,358,240	6,543,983,377
	Ce _infested_s2	52,370,520	6,545,649,940
	Ce _infested_s3	52,438,000	6,554,081,886
	Ce _healthy_s1	52,288,720	6,535,422,738
	Ce _healthy_s2	52,503,860	6,562,309,030
	Ce _healthy_s3	52,482,800	6,559,684,488
	**Sum**	**314,442,140**	**39,301,131,459**
**Hemocytes**	He_infested_s1	52,267,960	6,533,044,876
	He_infested_s2	52,229,740	6,528,057,206
	He_infested_s3	52,419,880	6,551,818,232
	He_healthy_s1	52,557,600	6,569,029,380
	He_healthy_s2	52,356,000	6,543,836,808
	He_healthy_s3	52,520,780	6,564,434,452
	**Sum**	**314,351,960**	**39,290,220,954**

Table S2: Statistics for the *de novo*-assembled transcripts of *M. yessoensis*.

	All	≥500bp	≥1000bp	N50	Total Length	Max Length	Min Length	Average Length
**Unigene**	80,831	65,095	38,843	2632	1.3E+08	40,012	301	1607

Table S3: Statistics for the annotation of the assembled transcripts of *M. yessoensis* with different databases.

Database	NR	SWISSPROT	KOG	GO	KEGG
**Annotation_numbers**	25,319	19,201	16,387	17,567	7,253
**Annotation_ratio**	31.32%	23.75%	20.27%	21.73%	8.97%

Supplementary Figures

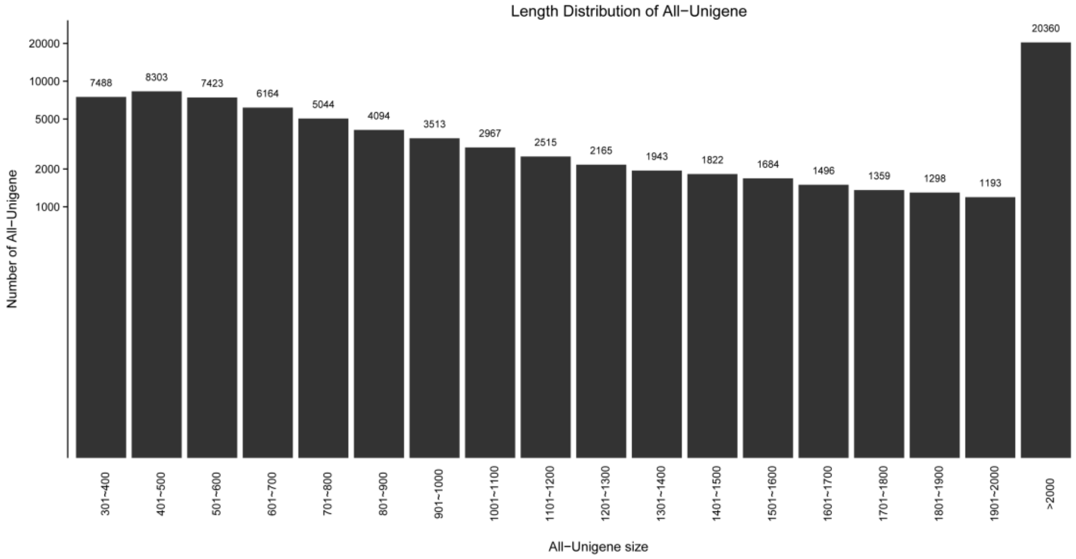

Figure S1. Length distribution of the *de novo* assembled unigenes.

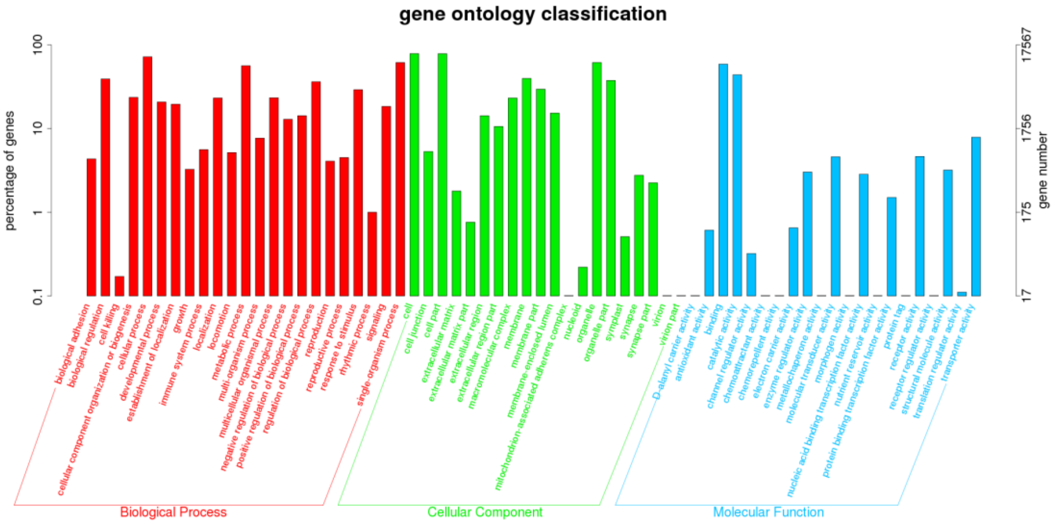

Figure S2. The GO annotation of unigenes in the transcriptomes.

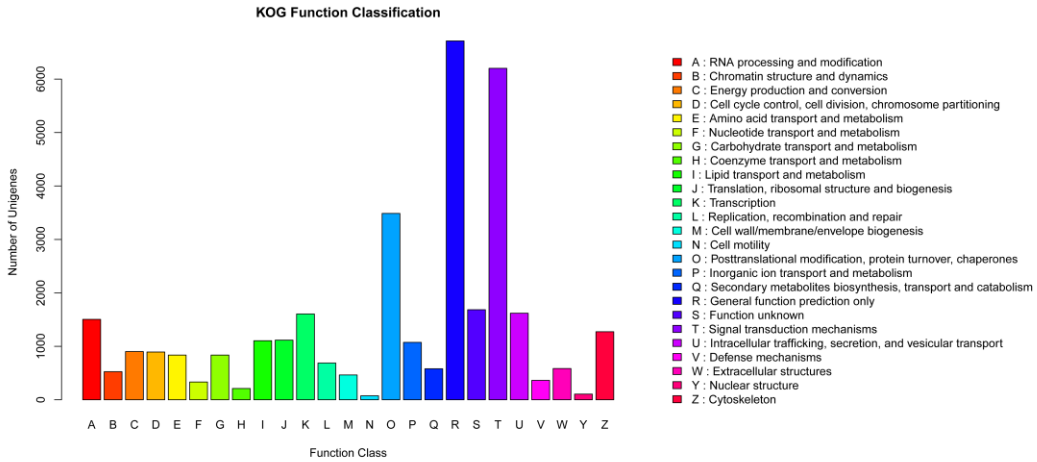

Figure S3. The KOG classifications of the assembled unigenes.

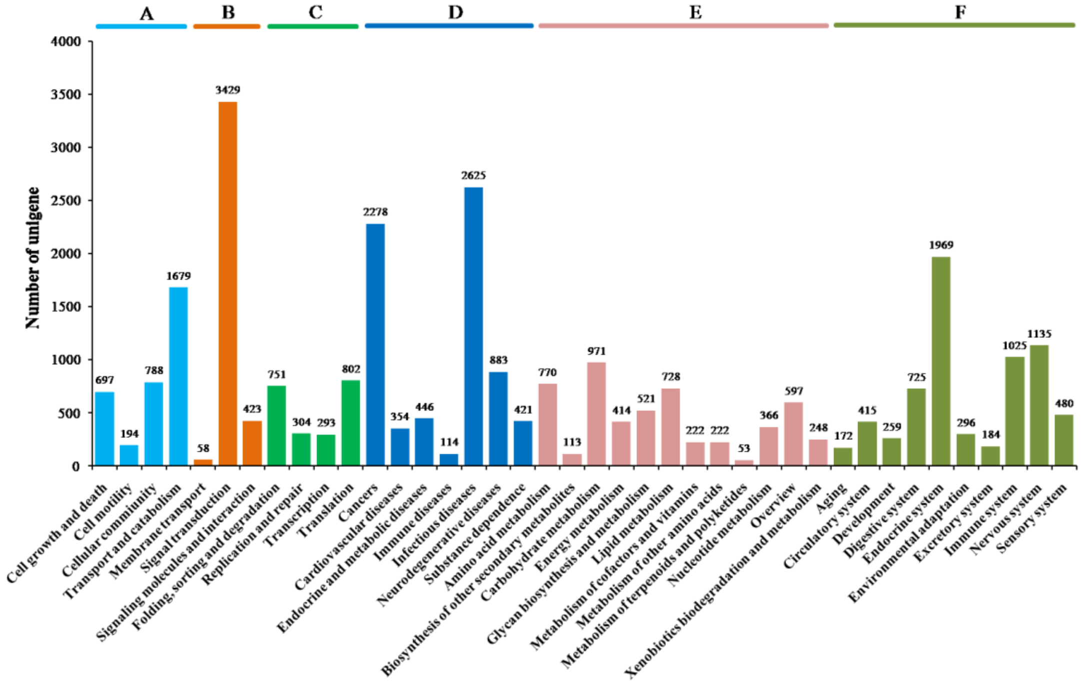

Figure S4. The KEGG classifications of the assembled unigenes. (A) cellular processes, (B) environmental information processing, (C) genetic information processing, (D) human diseases, (E) metabolism, (F) organismal systems.

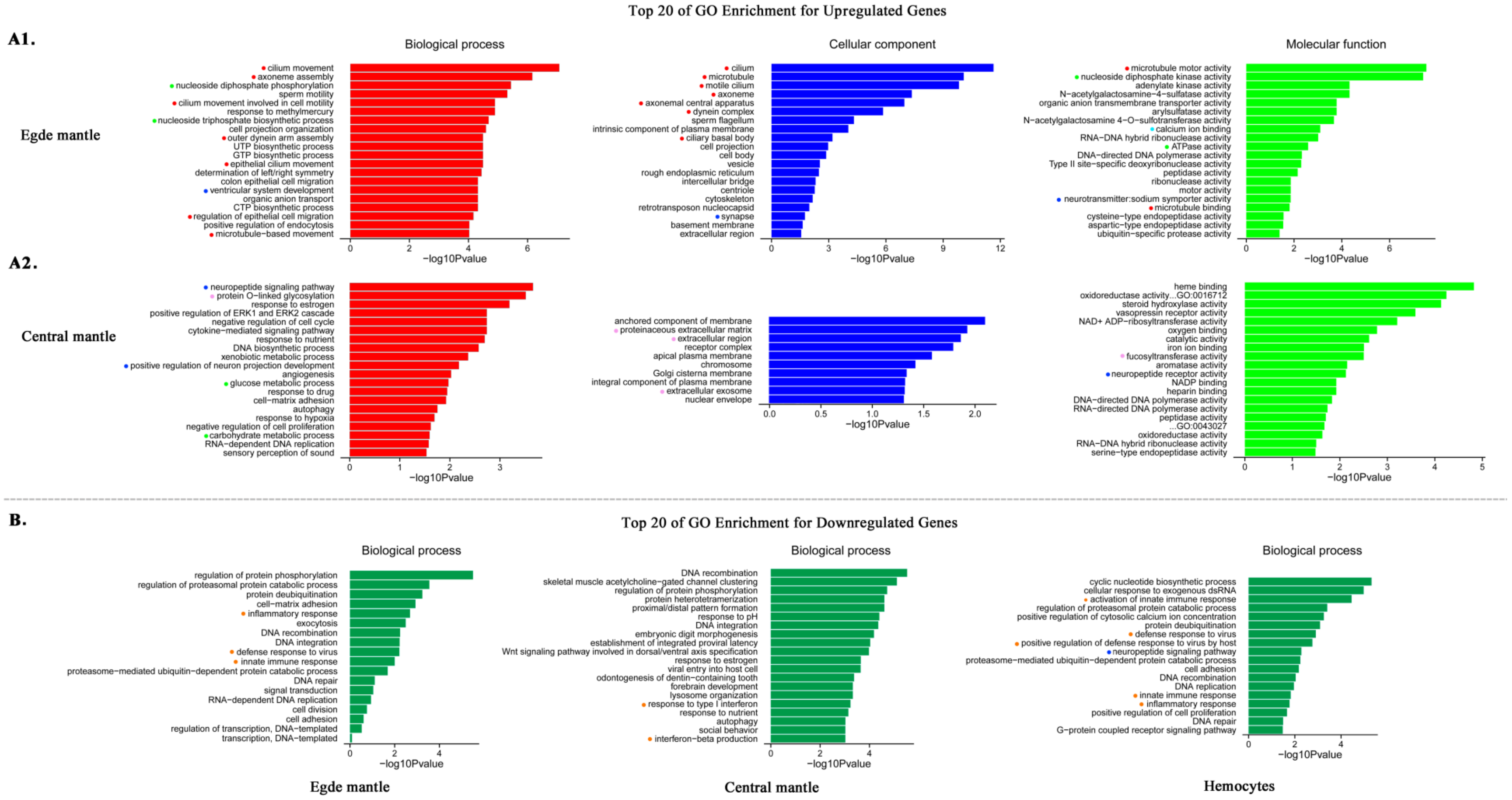

Figure S5. GO terms significantly enriched among up (A1 and A2) and downregulated (B) unigenes in the edge mantle, central mantle and hemocytes, respectively.

Only the top 20 terms are presented, which were sorted by the p-value ($p \leq 0.05$) and displayed with the $-\log_{10}$(p-value). Terms related to ‘cilium assembly and movement’ are highlighted with red dots, ‘glycometabolism metabolism’ with green dots, ‘nervous system’ with deep blue dots, ‘calcium binding or transport’ with light blue dots, ‘shell organic matrix with pink dots and ‘immune response’ with orange dots.
